# Prevalence of Cardiac Amyloidosis in Patients Undergoing Echocardiography at a Tertiary Center

**DOI:** 10.1016/j.jacadv.2024.101581

**Published:** 2025-01-23

**Authors:** Jonathan D. Knott, Rana Rashwan, Christopher G. Scott, Martha Grogan, Patricia A. Pellikka

**Affiliations:** a Department of Cardiovascular Medicine, Mayo Clinic, Rochester, Minnesota, USA; b Department of Quantitative Health Sciences, Mayo Clinic, Rochester, Minnesota, USA

**Keywords:** AL cardiac amyloidosis, ATTR cardiac amyloidosis, cardiac amyloidosis, echocardiography, heart failure

Cardiac amyloidosis (CA) is an increasingly recognized cause of heart failure worldwide.^1^ Once considered a rare disease, advancements in noninvasive diagnostic testing have led to increased diagnosis.^1^^,^^2^ However, CA likely remains underdiagnosed.^1^, ^2^, ^3^

**What is the clinical question being addressed?**

What is the prevalence of CA among adult patients undergoing clinically indicated echocardiography?

**What is the main finding?**

Of 31,027 patients undergoing echocardiography, 388 (1.25%) were found to have a diagnosis of CA; possible CA (inconclusive/unpursued confirmatory testing) was present in 165 (0.53%).

While recent data highlight important epidemiological trends,^3^^,^^4^ the prevalence of CA among patients referred for cardiac evaluation remains unknown. Echocardiography remains the cornerstone of this initial evaluation.^2^ Several “red flag” echocardiographic parameters have been suggested for CA, although are limited by lack of specificity, leading to delays in diagnosis.^2^ Echocardiographic machine-learning models to augment the detection of CA may allow for more widespread and earlier identification of CA.^5^ Development, testing, and clinical application of these models requires an estimation of pretest probability which relies on disease prevalence among those undergoing echocardiography; these data are currently lacking.

This retrospective, single-center, International Review Board-approved observational cohort study aimed to investigate the prevalence of both amyloid light chain (AL) and transthyretin amyloid (ATTR) CA in unselected adult patients undergoing a clinically indicated transthoracic echocardiogram at Mayo Clinic in Rochester, Minnesota from January 1 to December 31, 2022. We included patients who consented to inclusion in research. Patients with prior heart transplantation were excluded.

We utilized International Classification of Diseases (ICD)-10 codes to identify patients with systemic amyloidosis. We simultaneously screened all echocardiogram reports for the presence or possible presence of CA based on the interpreting physician’s description. Patients with ≥1 ICD-10 code and/or echocardiographic reports suggesting CA underwent further adjudication to confirm disease presence and classify disease type in accordance with the European Society of Cardiology guidelines.^1^

Patients with echocardiographic features of CA with inconclusive or unpursued confirmatory testing were defined as “possible CA.”

We calculated the prevalence of CA by dividing the total number of definite cases by the total number of patients within the cohort and reported the associated 95% exact binomial CIs. We stratified the prevalence of definite and possible CA by age group. Analyses were performed using SAS, version 9.4.

Both ICD codes and echocardiogram reports suggested CA in 376 patients with 280 (74%) adjudicated as definite and 58 (15%) as possible CA. Another 548 patients had an ICD code, although no echocardiogram report suggesting CA with 107 (20%) adjudicated as definite and 74 (14%) as possible CA. Echocardiographic impressions of CA without an ICD code were present in 75 with 1 (1%) adjudicated as definite and 33 (44%) as possible CA.

Of 31,027 patients (age 64 ± 16 years, 56% male), 388 (1.25%; 95% CI: 1.13-1.38) had a diagnosis of CA; 146 (0.47%; 95% CI: 0.40-0.55) and 237 (0.76%; 95% CI: 0.67-0.87) of these patients had diagnoses of AL and ATTR CA, respectively (Figure 1). Additional subtypes of CA included 1 patient with AA (inflammatory), 2 patients with apolipoprotein A, 1 patient with coexisting AL and ATTR, and 1 patient with unidentified CA. Possible CA was present in 165 (0.53%; 95% CI: 0.45-0.62) patients.

**Figure 1:**
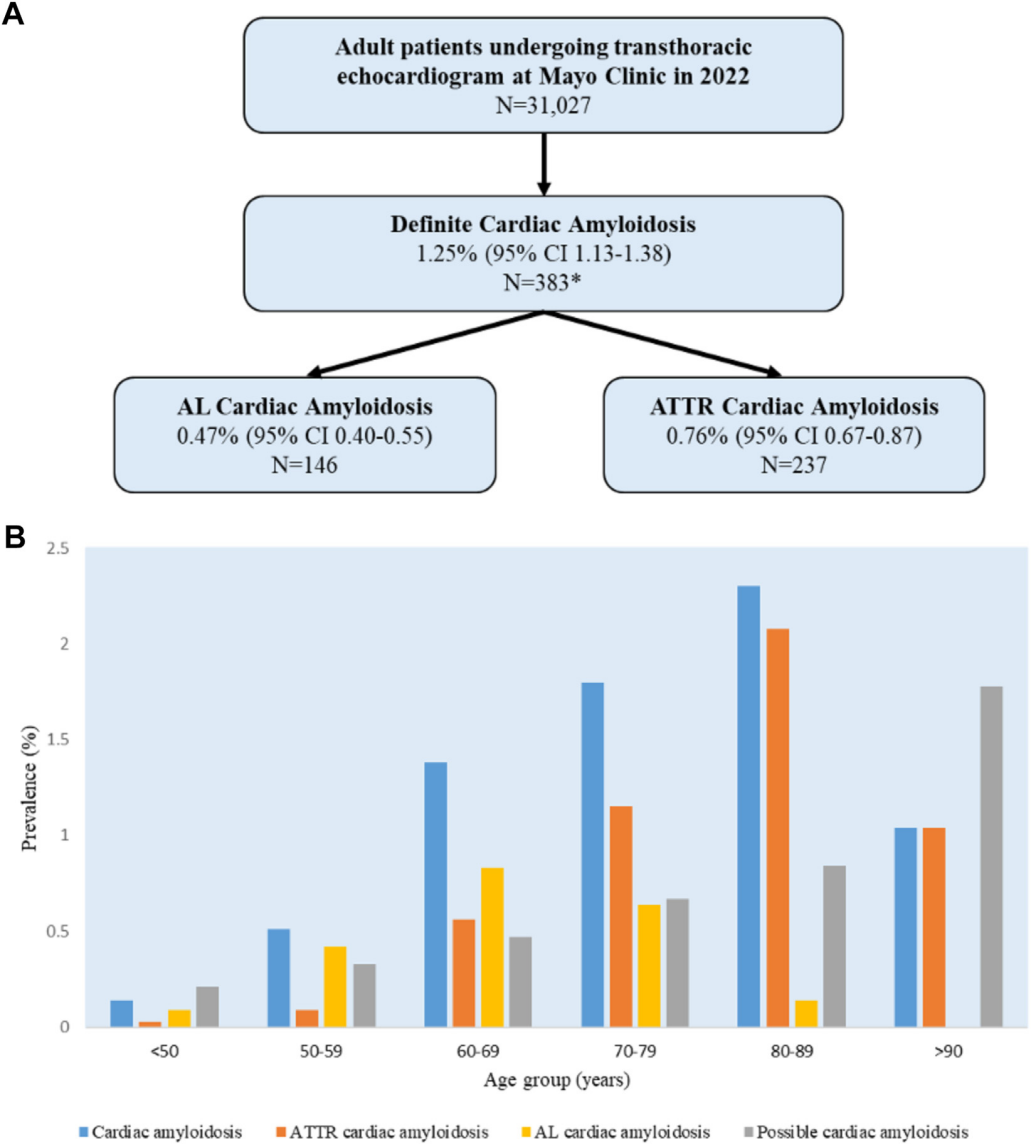
Prevalence of Cardiac Amyloidosis in Patients Undergoing Echocardiography and Stratification by Age Group (A) Prevalence of cardiac amyloidosis in patients undergoing echocardiography (B) Stratified by age group. ∗5 had additional subtypes of CA. AL = amyloid light chain; ATTR = transthyretin amyloid; CA = cardiac amyloidosis.

Stratified by age group, those with CA included 8 (0.14%; 95% CI: 0.06-0.27) patients <50 years of age, 23 (0.51%; 95% CI: 0.33-0.77) 50 to 59 years of age, 107 (1.38%, 95% CI: 1.12-1.64) 60 to 69 years of age, 147 (1.80%; 95% CI: 1.51-2.09) 70 to 79 years of age, 96 (2.30%; 95% CI: 1.87-2.80) 80 to 89 years of age, and 7 (1.04%; 95 CI: 0.42-2.12) >90 years of age (Figure 1).

This represents the first U.S. analysis evaluating the prevalence of CA among adults undergoing transthoracic echocardiography. The overall prevalence of CA in our adjudicated cohort was 1.25% with a higher prevalence of ATTR (0.76%) than AL (0.47%) CA. CA (AL and ATTR) was most common in the 80- to 89-year age group. ATTR CA had its peak prevalence in older patients (80-89 years of age) compared to AL (60-69 years of age). Another 0.53% had echocardiographic features of CA but testing to confirm the diagnosis was incomplete or not pursued.

CA remains significantly underdiagnosed and epidemiological data regarding its prevalence among those referred for cardiac evaluation are limited. Gilstrap et al evaluated hospitalized Medicare beneficiaries aged ≥65 years of age from 2002 to 2012 and found an overall prevalence of 55.2 per 100,000 person-years in 2012. Both the prevalence and incidence of CA had significantly increased since 2002, particularly in men, Black patients, and those ≥75 years of age.^3^ The authors did not discriminate between AL and ATTR CA but relied on a combination of ICD-9 codes for both systemic amyloidosis and heart failure. It is likely that these limitations, in addition to the selected population of inpatients only, led to an underestimation of the true prevalence of disease.

Determining the prevalence of CA in patients referred for echocardiography is important as testing the diagnostic accuracy of imaging parameters is impacted by the pretest probability of disease. We report the first U.S. data using verified diagnoses in this population showing a 1.25% disease prevalence which is similar to an Italian prospective study of 5,315 patients ≥55 years of age referred for an echocardiogram. They reported a similar age-distribution of CA subtypes with the highest prevalence in those >80 years of age.^4^ Our data complement these findings and are important to strategies for improved screening and earlier detection of CA.

As there are no specific ICD-10 codes for CA, we may have underestimated disease prevalence. Data were obtained from a single tertiary center and referral bias may affect the observed prevalence rates.

## Funding support and author disclosures

Dr Pellikka receives research support from Ultromics, with money paid to her institution; is supported as the Betty Knight Scripps- George M. Gura, Jr. MD, Professor of Cardiovascular Diseases Clinical Research, Mayo Clinic. All other authors have reported that they have no relationships relevant to the contents of this paper to disclose.
